# Enteral Sodium Chloride Supplementation and Fluid Balance in Children Receiving Diuretics

**DOI:** 10.3390/children9010094

**Published:** 2022-01-11

**Authors:** Laura Ortmann, Teri J. Mauch, Jean Ballweg

**Affiliations:** 1 Department of Pediatrics, University of Nebraska Medical Center, Omaha, NE 68198, USA; tmauch@childrensomaha.org (T.J.M.); jballweg@childrensomaha.org (J.B.); 2 Children’s Hospital & Medical Center, Omaha, NE 68114, USA

**Keywords:** hyponatremia, fluid overload, diuretics, hypochloremia, pediatric

## Abstract

The use of sodium chloride (NaCl) supplementation in children being prescribed diuretics is controversial due to concerns that supplementation could lead to fluid retention. This is a single-center retrospective study in which fluid balance and diuretic dosing was examined in children prescribed enteral NaCl supplements for hyponatremia while receiving loop diuretics. The aim of this study was to determine whether significant fluid retention occurred with the addition of NaCl. Fifty-five patients with 68 events were studied. The median age was 5.2 months, and 82% were hospitalized for cardiac disease. Daily fluid balance the seven days prior to NaCl supplementation was lower than the seven days after, with measurement of: median 17 mL/kg/day (7–26) vs. 22 mL/kg/day (13–35) (*p* = 0.0003). There was no change in patient weight after supplementation (*p* = 0.63). There was no difference in the median loop diuretic dose before and after supplementation, with the diuretic dose in furosemide equivalents of 3.2 mL/kg/day (2.3–4.4) vs. 3.2 mL/kg/day (2.2–4.7) (*p* = 0.50). There was no difference in the proportion of patients receiving thiazide diuretics after supplementation (56% before vs. 50% after (*p* = 0.10)). NaCl supplementation in children receiving loop diuretics increased calculated fluid balance, but weight was unchanged, and this was not associated with an increase in diuretic needs, suggesting clinicians did not consider the increase in fluid balance to be clinically significant.

## 1. Introduction

Fluid overload is common in hospitalized children, occurring in over 30% of patients admitted to the pediatric intensive care unit (PICU) [1,2]. This can be due to aggressive fluid resuscitation, endothelial dysfunction leading to capillary leak, acute kidney injury, or neuro-endocrine activation. The ability to restrict fluid intake can be limited due to the need to provide adequate nutrition. Therefore, diuretics are often used to enhance urine output and decrease fluid balance.

Loop diuretics are typically the first line of therapy for fluid overload and work by blocking sodium resorption in the thick ascending limb of the loop of Henle, causing sodium loss in the urine, which is followed by water [3]. Thiazide diuretics are often added to complement loop diuretics and also result in sodium loss [4,5]. Hyponatremia is a known side effect of diuretic use [6,7]. Severe hyponatremia can lead to cerebral injury, and children that develop hyponatremia during hospitalization are at increased risk for seizures and death [8,9]. Thus, clinicians try to avoid significant hyponatremia, even when giving medications that waste sodium by design.

Knowing how to approach diuretic-induced hyponatremia can be difficult. Sodium chloride (NaCl) supplementation is commonly ordered for children on diuretics [7] but is controversial due to concerns that sodium use will exacerbate fluid retention. How NaCl supplements affect fluid retention and fluid balance in children is unknown. The aim of this study was to evaluate the effect of enteral NaCl supplementation on fluid balance, weight, and diuretic need in hospitalized children receiving loop diuretics. We hypothesized that NaCl supplementation used to maintain physiologic plasma sodium levels would not increase fluid balance or diuretic needs. This was based on the theory that normal sodium levels would decrease neuro-endocrine activation and improve diuretic effectiveness. 

## 2. Materials and Methods

This study was approved by the Institutional Review Board of the University of Nebraska Medical Center (0216-18-EP). We performed a retrospective chart review of children less than 18 years of age admitted to the Children’s Hospital & Medical Center PICU or med/surg floors from 1 January 2015 to 31 December 2017. Children with all diagnoses were eligible for inclusion if they received scheduled or a continuous infusion of loop diuretics for at least 7 days without NaCl supplementation, then received scheduled enteral NaCl for a minimum of 7 days while inpatient in order to compare fluid outcomes before and after supplementation. Children were excluded if they were receiving renal replacement therapy or were discharged less than 7 days after starting NaCl supplementation. In cases where a child had NaCl supplementation discontinued for at least 14 days then reordered, the second instance was analyzed as a separate supplementation event.

Presence of acute kidney injury was determined using the Kidney Disease Improving Global Outcomes criteria [10]. Children were classified as post-operative if they underwent surgery prior to NaCl supplementation during the same hospital stay. Diagnosis of heart failure was determined by the clinical team.

Daily fluid balance was calculated the 7 days before and the 7 days after NaCl supplementation was started. The day NaCl was first ordered was not included in fluid calculations. Daily fluid balance was calculated from the difference between the total fluid intake and the total fluid output. Change in weight was also recorded the 7 days before and after the start of NaCl supplementation as another measure of fluid balance [11]. Total loop diuretic dose in IV furosemide equivalents was calculated before and after NaCl supplementation using the following conversions: 1 mg IV/enteral bumetanide = 40 mg IV furosemide; 1 mg enteral furosemide = 0.5 mg IV furosemide [12,13]. Use of thiazide diuretics was also recorded to see if changes in second-line diuretics occurred with NaCl supplementation. To test the effect of plasma sodium level on fluid balance and diuretic need, subgroup analysis was performed on patients with a sodium level <130 mmol/L vs. those with a sodium level ≥130 mmol/L on the day NaCl supplementation was started.

Fluid intake and output, urine output, and weight were compared for the seven days before and the seven days after NaCl supplementation. Data were collected in a REDCap database [14]. Categorical data were reported as numbers and percentages, and continuous data as medians (interquartile range) or mean ± standard deviation with normality being determined with the Shapiro–Wilk test. Differences in plasma sodium and chloride levels were analyzed using the paired t-test. Fluid balance and diuretic use before and after NaCl supplementation were compared using the Wilcoxon signed-rank test for paired samples and Mann–Whitney U for independent samples. McNemar’s test was used to compare the incidence of thiazide diuretic use before and after NaCl supplementation. Statistical significance was set at <0.05. All tests were 2-sided and performed using SAS Studio 3.6 (SAS Institute, Cary, NC, USA).

## 3. Results

Fifty-six patients were included for a total of 68 supplementation events. The median age was 5.2 months (2.2–10.0) and median weight was 5.6 kg (4.4–8.0). Cardiac disease was the primary diagnosis in 82% of patients (Table 1). Three (5%) children had a prior history of acute kidney injury (AKI), though none met criteria for AKI when NaCl was ordered. 

The mean plasma sodium level at hospital admission was 137 ± 4 mmol/L (range 129–151). The mean plasma sodium level when NaCl was ordered was 130 ± 3 mmol/L (range 122–140), which was significantly different than sodium level at admission, *p* < 0.0001. Sodium level increased after seven days of NaCl supplementation to 135 ± 4 mmol/L (range 123–148), *p* < 0.0001. The mean chloride level on the day NaCl was ordered was 92 ± 5 mmol/L (range 78–104) and increased after supplementation to 98 ± 6 mmol/L (range 77–116), *p* < 0.0001. There was no difference in plasma sodium levels on the day NaCl was ordered between patients that received chlorothiazide or hydrochlorothiazide compared to those that did not (130 ± 3 mmol/L vs. 129 ± 3 mmol/L, *p* = 0.34),and no difference between those that received metolazone and those that did not (130 ± 3 mmol/L vs. 130 ± 4 mmol/L, *p* = 0.79) prior to supplementation. The median starting NaCl dose was 2.5 mEq/kg/day (IQR 1.8–3.0). 

The median daily fluid balance and diuretic dose the seven days before and the seven days after starting NaCl are shown in Table 2, demonstrating an increase in measured fluid balance, but no change in weight. There was no change in urine output after NaCl supplementation. 

There was no change in loop diuretic dosing after supplementation (Table 2). Chlorothiazide or hydrochlorothiazide was used in 56% of patients before starting NaCl supplementation and 50% after (*p* = 0.10); metolazone use was 37% before and 25% after (*p* = 0.10). Use of mineralocorticoid-receptor antagonists was similar before and after supplementation (34% vs. 35%, *p* = 0.85).

Patients whose sodium level was <130 mmol/L on the day NaCl was started were compared to those whose sodium level was ≥130 mmol/L to examine differences in outcomes based on sodium level. The groups were similar in age (3.5 vs. 6.5 months, *p* = 0.32) and loop diuretic dosing before supplementation (3.2 vs. 3.5 mg/kg/day, *p* = 0.36). The sodium <130 group had an increase in fluid balance, with no changes in urine output, weight, or loop diuretic dose (Table 3). 

**Figure 1:**
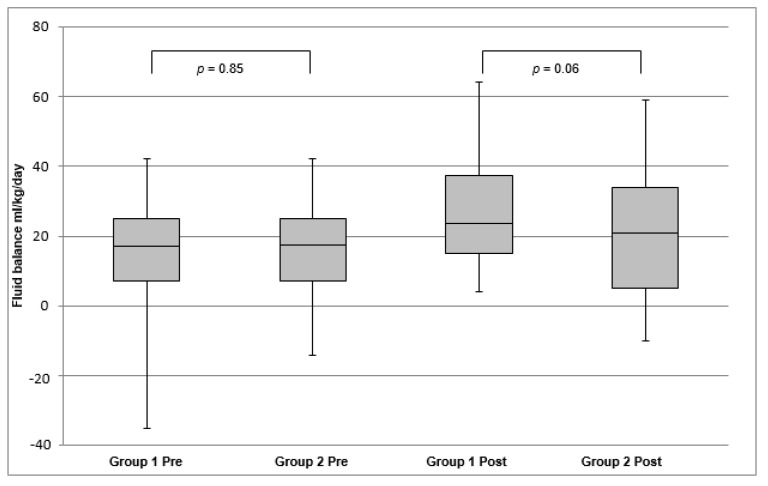
Average daily fluid balance before and after starting NaCl supplementation.

The sodium ≥ 130 group did not have a statistically significant difference in fluid balance, weight, or urine output, and loop diuretic dose was unchanged. There was no significant difference between the <130 and the ≥130 groups in pre or post supplementation fluid balance (Figure 1).

There were no adverse neurologic events reported secondary to hyponatremia. The average length of hospital stay was 83 days (IQR 42–139), and there was no correlation between the admission sodium level [r(61) = 0.03] or sodium level at the start of supplementation [r(61) = −0.14] with length of stay. Survival to hospital discharge was 84%. There was no difference between survivors and non-survivors in the admission sodium level (137 ± 4 mmol/L vs. 136 ± 4 mmol/L, *p* = 0.29) or level at the start of NaCl supplementation (130 ± 3 mmol/L vs. 130 ± 3 mmol/L, *p* = 0.91).

## 4. Discussion

This study found an increase in fluid balance, but no increase in weight, urine output, or diuretic dose, in children receiving diuretics started on enteral NaCl supplementation. There was an increase in enteral feeds with no change in urinary output. Diuretics were not increased in conjunction with the increasing fluid balance, suggesting that the medical team did not view the increase in fluid balance as clinically significant. Sodium levels improved or remained unchanged in 93% of supplementation events. Normalizing sodium levels can not only prevent critical adverse events from hyponatremia but may be beneficial by decreasing renin–angiotensin–aldosterone system (RAAS) activation, improving diuretic efficacy and improving infant growth. There may also be a benefit to normalizing chloride levels.

While we cannot determine the exact etiology of hyponatremia in individual patients, the cause is likely multifactorial and may not simply be a result of diuretic-induced sodium wasting. The majority of children in this study were hospitalized with cardiac disease (82%). The RAAS is activated both after cardiac surgery and with heart failure [15,16,17,18]. RAAS activation leads to absorption of urinary sodium and water in order to increase the circulating blood volume and thus improve organ perfusion pressure. This leads to fluid overload and may prompt the ordering of diuretics. Diuretics stimulate RAAS activation [19,20,21]. This potentially sets up a cycle where the body attempts to absorb sodium and water to maintain perfusion in the setting of underlying disease and forced diuresis, while the medical team orders more diuretics to counter the fluid overload worsened by RAAS activation. 

Increasing serum sodium levels has the potential to decrease RAAS activation [22,23]. Sodium and chloride are also required for diuretic function. Early studies of loop diuretics found that the diuretic effect was significantly influenced by sodium balance, with sodium depletion decreasing natriuresis [24]. This has led to the use of hypertonic saline infusions to augment diuresis and overcome diuretic resistance. In adults with heart failure infusions of hypertonic saline in conjunction with loop diuretics improves urine output, heart failure symptoms, and long-term outcomes [25,26,27]. However, we did not see an increase in urine output after starting NaCl supplementation.

Sufficient sodium is necessary for infant growth, and milk alone may not provide enough in infants who are wasting sodium due to diuretic use. Sodium supplementation has been found to improve weight gain in premature infants without an increase in edema [28,29,30]. Our study did not involve the neonatal intensive care unit and thus did not include premature infants, but our cohort represents a medically fragile population. Sodium may also be vital for the growth of these children. 

Another likely cause for hyponatremia in this cohort was the increased delivery of free water. The median age in this study was 5 months, indicating that a majority of children were given breast milk or formula for enteral nutrition. Over the fifteen-day study period there was a transition from intravenous fluids (IVF) and total parenteral nutrition (TPN) to enteral hydration and nutrition. By the end of the study period all children were receiving most of their fluids from enteral nutrition. For a child receiving the median post-supplementation feeds (106 mL/kg/day), they would obtain approximately 0.7 mEq/kg/day of sodium from their feeds [31,32]. This is substantially less than what is typically given in IVF or TPN (a child receiving maintenance fluids of ½ NS will receive approximately 7.7 mEq/kg/day of sodium). With sodium wasting diuretics being continued during this IVF/TPN-to-feeding transition, hyponatremia is not surprising. That enteral feeds were associated with hyponatremia is also supported by the fact that the group with sodium levels <130 mmol/L had a greater intake of feeds compared to those with a sodium ≥130 mmol/L.

NaCl supplements do not just provide sodium. Chloride is the second most common electrolyte in the plasma and plays an important role in acid–base balance and sodium homeostasis. It is wasted along with sodium in diuretic use but has been studied far less. A majority of subjects were also hypochloremic. Hypochloremia has been linked to increased cardiovascular mortality in adults [33,34] and may be associated with diuretic resistance [35]. Studies in children disagree on whether hypochloremia is associated with worse outcomes and are limited by small sample size and low mortality [36,37,38]. Chloride supplements are given to hospitalized children with hypochloremic metabolic alkalosis [39,40], but data are lacking on whether supplementation improves outcomes. Further investigation is warranted into hypochloremia’s impact on pediatric illness and whether normalizing chloride levels improves outcomes.

This study was limited by its retrospective and single-center design and the lack of a control group. There is no standard practice in this institution for the treatment of mild to moderate hyponatremia, so there was considerable variation in the timing of supplementation and the dose ordered. Given local practice, we were unable to build a historic control group that had hyponatremia but were not started on NaCl supplementation. Clinicians may also choose to reduce diuretic use in the face of hyponatremia, even with a positive fluid balance. With the limitations in the electronic medical record, it is not always possible to determine whether diuretics are being decreased due to patient improvement or medication side effects. A prospective randomized study of NaCl supplementation to treat hyponatremia associated with diuretic use would be ideal, but given the potential consequences of hyponatremia, equipoise may be lacking.

Total sodium provided by IVF/TPN was not collected due to the high number of infusions many of these patients were on during the study period, so the assumption that the sodium intake decreased as feeds were increased could be incorrect. However, given the low sodium content of infant feeds and the move away from hypotonic fluids in pediatrics [41], it is highly likely that children were receiving less sodium by the end of the study period. The majority of the cohort were children with heart disease, as these are the ones most likely to require scheduled diuretics. The non-cardiac patient population was too small to perform a subanalysis and may not respond the same way to NaCl supplementation.

## 5. Conclusions

NaCl supplementation increased measured fluid balance but did not increase weight in children receiving diuretics. Diuretic dose did not change in response to the increased fluid balance. Further investigations with a control group would aid in determining whether NaCl supplementation alters outcomes.

## Figures and Tables

**Table 1:** Patient demographic data.

	Number (%) n = 56
Admission diagnosis category	
Cardiac—single ventricle post-	
operative	20 (36)
Cardiac—two ventricle post-operative	9 (16)
Cardiac—heart failure	8 (14)
Cardiac—other	9 (16)
Non-cardiac—respiratory	4 (7)
Non-cardiac—other	6 (11)
Sex, male	32 (58)
Supplementation event variables	Number (%) n = 68
Location of first NaCl order	
PICU	47 (69)
Med Surg	21 (31)
On vasoactive support	19 (28)
On mechanical ventilation	29 (43)

**Table 2:** Fluid balance and diuretic dose before and after NaCl supplementation.

Variable	7 Days Prior to Starting NaCl	7 Days after Starting NaCl	*p* Value
Fluid balance, mL/kg/day	17 (7–26)	22 (13–35)	0.0003
Total fluid in, mL/kg/day	122 (104–136)	130 (104–152)	0.0002
Enteral feeds in, mL/kg/day	75 (52–98)	106 (88–125)	<0.0001
TPN in, mL/kg/day	0 (0–28.6)	0 (0–0.2)	<0.0001
IVF in, mL/kg/day	23 (10–38)	8 (0.8–23)	<0.0001
Total fluid out, mL/kg/day	106 (88–118)	103 (88–123)	0.90
Urine output, mL/kg/hour	4.0 (3.1–4.7)	3.9 (3.2–4.5)	0.62
Surgical drain out, mL/kg/day	0 (0–2.6)	0 (0–0)	0.10
Weight change, kg	0 (−0.2–0.2)	0 (−0.1–0.2)	0.63
Loop diuretic, mg/kg/day	3.2 (2.3–4.3)	3.2 (2.2–4.7)	0.50

**Table 3:** Fluid balance and diuretic need based on plasma sodium level.

Variable	7 Days Prior to NaCl	7 Days after NaCl	*p* Value
Fluid balance, ml/kg/day			
Sodium < 130	17 (7–25)	24 (15–37)	0.0002
Sodium ≥ 130	17.5 (7–26)	21 (5–34)	0.23
Enteral feeds, mL/kg/day			
Sodium < 130	89 (57–107)	115 (90–136)	<0.0001
Sodium ≥ 130	72 (50–82)	101 (84–119)	<0.0001
Weight change, kg			
Sodium < 130	0 (−0.2–0.3)	0.1 (−0.1–0.2)	0.99
Sodium ≥ 130	0.1 (−0.1–0.2)	0.1 (−0.1–0.3)	0.57
UOP, mL/kg/hour			
Sodium < 130	4.0 (3.1–4.7)	4.0 (3.4–4.5)	0.65
Sodium ≥ 130	4.1 (3.1–4.8)	4.1 (2.8–4.7)	0.50
Loop diuretics, mg/kg/day			
Sodium < 130	3.2 (2.2–3.8)	2.7 (2.0–4.1)	0.97
Sodium ≥ 130	3.5 (2.6–4.4)	3.2 (2.3–5.0)	0.37

## Data Availability

Data will be made available upon reasonable request.
